# Potent Antimicrobial Azoles: Synthesis, In Vitro and In Silico Study

**DOI:** 10.3390/antibiotics13111044

**Published:** 2024-11-04

**Authors:** Zeynep Özdemir, Yaren Nur Zenni, Arzu Karakurt, Suat Sari, Selma Saraç, Mevlüt Akdağ, İrem Bozbey Merde, Didem Kart, Roberto Venanzoni, Giancarlo Angeles Flores, Paola Angelini, Muzammil Kabier, Bijo Mathew, Simone Carradori

**Affiliations:** 1 Department of Pharmaceutical Chemistry, Faculty of Pharmacy, Inonu University, 44280 Malatya, Türkiye; 2 Department of Pharmaceutical Chemistry, Faculty of Pharmacy, Lokman Hekim University, 06100 Ankara, Türkiye; 3 Department of Pharmaceutical Chemistry, Faculty of Pharmacy, Hacettepe University, 06100 Ankara, Türkiye; 4 Department of Pharmaceutical Chemistry, Faculty of Pharmacy, Baskent University, 06790 Ankara, Türkiye; 5 Department of Pharmaceutical Chemistry, Faculty of Pharmacy, Afyonkarahisar Health Sciences University, 03030 Afyonkarahisar, Türkiye; 6 Department of Pharmaceutical Microbiology, Faculty of Pharmacy, Hacettepe University, 06100 Ankara, Türkiye; 7 Department of Chemistry, Biology and Biotechnology, University of Perugia, 06123 Perugia, Italy; 8 Department of Pharmaceutical Chemistry, Amrita School of Pharmacy, Amrita Vishwa Vidyapeetham, AIMS Health Sciences Campus, Kochi 682 041, India; 9 Department of Pharmacy, “G. d’Annunzio” University of Chieti-Pescara, 66100 Chieti, Italy

**Keywords:** (arylalkyl)azole, imidazole, oxime ether, antifungal activity, molecular modelling, dermatophytes

## Abstract

**Background/Objectives**: The increase in fungal infections, both systemic and invasive, is a major source of morbidity and mortality, particularly among immunocompromised people such as cancer patients and organ transplant recipients. Because of their strong therapeutic activity and excellent safety profiles, azole antifungals are currently the most extensively used systemic antifungal drugs. Antibacterial properties of various topical antifungals, such as oxiconazole, which features oxime ether functionality, were discovered, indicating an exciting prospect in antimicrobial chemotherapy. **Methods**: In this study, eleven new oxime ether derivatives with the azole scaffold (**5a**–**k**) were synthesized and tested for their antimicrobial effects using the microdilution method to obtain broad-spectrum hits. **Results**: Although the title compounds showed limited efficacy against *Candida* species, they proved highly effective against dermatophytes. Compounds **5c** and **5h** were the most potent derivatives against *Trichophyton mentagrophytes* and *Arthroderma quadrifidum*, with minimum inhibitory concentration (MIC) values lower than those of the reference drug, griseofulvin. The MIC of **5c** and **5h** were 0.491 μg/mL and 0.619 μg/mL against *T. mentagrophytes* (MIC of griseofulvin: 2.52 μg/mL). The compounds were also tested against Gram-positive and Gram-negative bacteria. Briefly, **5c** was the most active against *Escherichia coli* and *Bacillus subtilis*, with MIC values much better than that of ciprofloxacin (MIC of **5c** = 1.56 μg/mL and 1.23 μg/mL, MIC of ciprofloxacin = 31.49 and 125.99 μg/mL, respectively). Molecular docking suggested a good fit in the active site of fungal lanosterol 14α-demethylase (CYP51) and bacterial FtsZ (Filamenting temperature-sensitive mutant Z) protein. **Conclusions**: As a result, the title compounds emerged as promising entities with broad antifungal and antibacterial effects, highlighting the utility of oxime ether function in the azole scaffold.

## 1. Introduction

Studies to produce strong and efficient antimicrobial chemicals are vital not only for controlling serious infections, but also for preventing and treating infections secondary to other therapies such as cancer and surgical operations [1,2]. Among the pharmacophore groups responsible for antimicrobial activity, azole rings play a crucial role in the development of more effective and broad-spectrum antimicrobials [3,4,5]. A number of compounds with azole groups have antibacterial and antifungal properties, according to the literature [6]. Beyond *Candida* species, dermatophytes are highly common as causative agents of more than 60% of fungal infections worldwide. Moreover, the emergence of antifungal-resistant dermatophytes has been reported, alarmingly [7].

**Figure 1:**
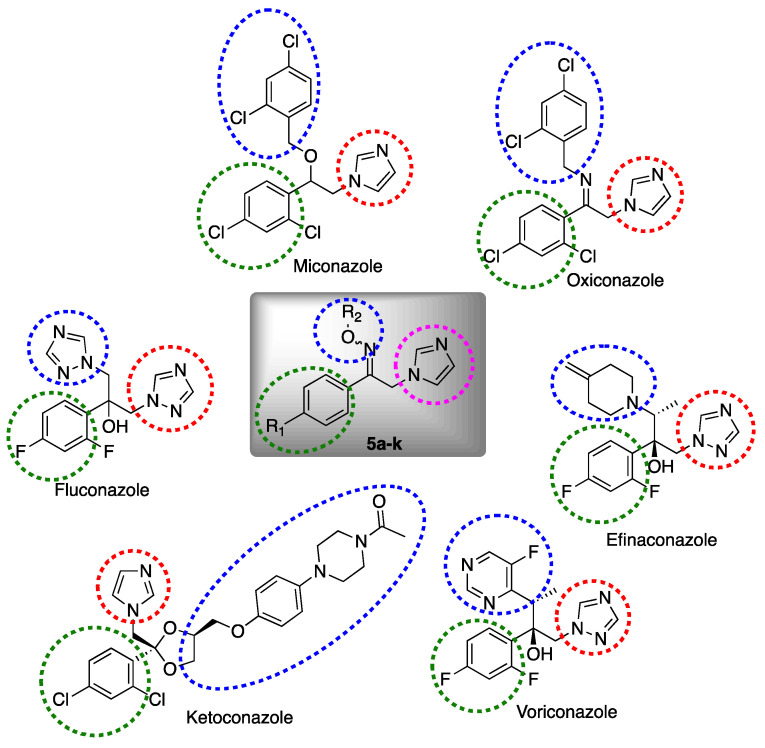
Main skeleton of azole antifungals. Group A is highlighted by a red circle, group B by a green circle, and group C group by a blue circle.

Azole antifungal drugs are the first choice in antifungal chemotherapy due to their broad spectrum, oral availability and tolerability [8]. Azoles inhibit fungal lanosterol 14α-demethylase (CYP51), preventing ergosterol production in fungal cell membranes, which leads to membrane disruption and the inhibition of fungal growth [9,10,11,12]. Azole antifungals share a common structural skeleton consisting of an azole ring (A group), an aromatic ring (B group), an ethylene bridge that connects these two rings, and a tail (C group) linked to this bridge from the carbon to which the aromatic ring is attached (C1) (Figure 1). In drugs like fluconazole and voriconazole, a hydroxyl group linked to the same C1 is hypothesized to make a water-mediated hydrogen bond with the side chain of a neighbouring tyrosine residue (e.g., *C. albicans* Tyr132) [13,14,15,16]. The azole ring blocks the heme co-factor of CYP51 by chelating with the iron, which catalyzes the oxidation process, whereas the aryl and tail groups fill the active site and provide tight binding. Thus, by interfering with fungal cell membrane permeability and the functioning of particular cell membrane enzymes, essential components in the cytoplasm are lost, and fungal growth is inhibited [13,14].

Azole antifungals are classified as imidazole and triazole derivatives, although one tetrazole derivative was recently approved for clinical use [17]. Imidazole derivatives are early members of this class, used mainly against topical mycoses, of which oxiconazole is an oxime ether derivative and effective against many dermatophytes [18]. Imidazole antifungals are also known for their antibacterial activity. Oxiconazole, miconazole, econazole and ketoconazole were reported for their potent effects against Gram-positive bacteria too, especially *Staphylococcus aureus* [19,20,21]. A combination of antifungal and antibacterial compounds was also demonstrated to be an effective approach, as in Azevedo et al.’s study where fluconazole’s MIC was reduced up to 100-fold against azole-resistant *Candida albicans* when combined with tetracycline or doxycycline [22].

Previously, we reported several azole derivatives with oxime ether moiety showing highly potent anti-*Candida* activity, some of which were also found to possess antibacterial effects [23,24,25,26]. In this study, novel oxime ether-derived compounds with an imidazole ring as the azole group and 4-methylphenyl and 4-trifluoromethylphenyl as the aryl group were synthesized in order to study their antimicrobial properties and discover dual-acting antifungal and antibacterial derivatives. To investigate the role of the tail group in activity, moieties with varying chain lengths, branching, unsaturation, and arylalkyl groups were incorporated into the oxime ether tail. Molecular modelling was applied to provide insights into their possible mechanism of action.

## 2. Results and Discussion

### 2.1. Chemistry

**Scheme 1:**
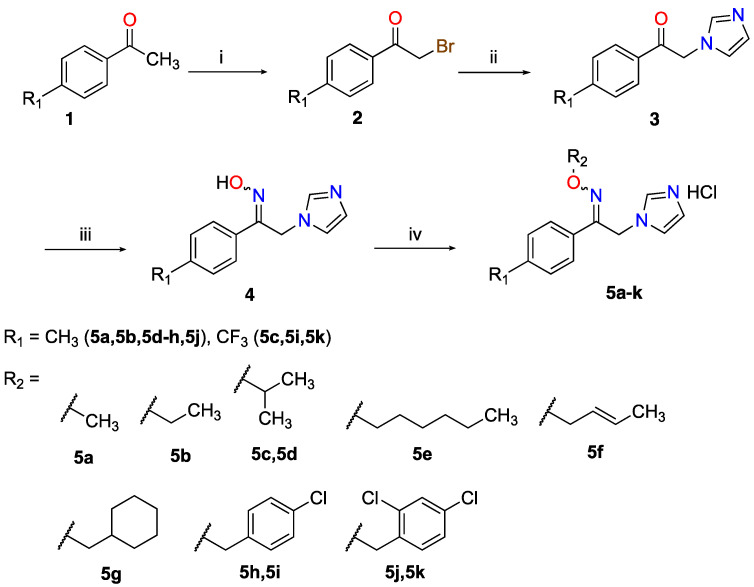
Synthesis and molecular structures of **5a**–**k**: (i) Br_2_, CH_3_COOH, 0–5 °C; (ii) imidazole, DMF, 0–5 °C to rt, overnight; (iii) NH_2_OH.HCl, C_2_H_5_OH, pH 14, reflux, 3 h; (iv) RX, DMF, rt, 2–6 h, gHCl.

Compounds **5a**–**k** were prepared according to Scheme 1. Their structures were confirmed using spectrum data from ^1^H-NMR, ^13^C-NMR, and TOF-MS (Supporting Information).

The initial chemicals, 2-bromo-*p*-tolylethanone and 2-bromo-*p*-trifluoromethylethanone, were produced by brominating 4-methylacetophenone/4-trifluoromethylacetophenone in an acetic acid solution with Br_2_. The N-alkylation reaction between 2-bromo-*p*-tolylethanone/2-bromo-*p*-trifluoromethylethanone and imidazole yielded 1-(4-methyl/trifluoromethylphenyl)-2-(1*H*-imidazol-1-yl)ethanone. In the procedure, a 2 mol excess of imidazole was utilized, ensuring a basic environment [23,24,26,27]. The compound was crystallized from methanol and ethyl acetate. The mechanism of the reaction was suggested to be bimolecular nucleophilic substitution (S_N_2), occurring first on the azole nitrogen atom, followed by the generation of the product by the moving of the double bond to the other nitrogen atom. At this point, the 4-methylphenyl derivative was acquired in a low yield of 50.2%, while the 4-trifluoromethyl derivative was procured in a much higher yield of 81.3%. The difference in yields most likely arises from the electron-withdrawing CF_3_ group, which further reduces the electron load of the carbon to which the bromine is attached. The treatment of the resultant ketone with hydroxylamine hydrochloride yielded 1-(4-methylphenyl)-2-(1*H*-imidazol-1-yl)ethanone oxime. In this condensation reaction, which uses the nucleophilic addition–elimination mechanism, hydroxylamine, whose nucleophilic power increases in a basic environment with a pH of 14 and a 15 N sodium hydroxide solution, attacks the carbonyl carbon of the ketone derivative, resulting in the addition reaction. The release of water from the molecule causes a double bond to form between carbon and nitrogen, occurring via an elimination reaction. The rate of oxime production is determined by the structure of the substrate, and at this stage, the 4-trifluoromethyl derivative was synthesized in a higher yield (68.3%).

In the synthesis of the target molecules, oxime ether derivatives, bases such as sodium ethoxide, sodium hydride, and potassium carbonate, are utilized to enhance proton separation in the oxime hydroxyl. Sodium ethoxide was used in this study. The initial attempts to synthesize oxime ether involved combining equimolar oxime and alkyl halide. The reaction was stirred and heated for 2–3 days; however, it was discovered that the starting material was not fully consumed. Increasing the quantity of alkyl halide thrice resulted in a completion time of 2–4 hours. The title compounds were purified via silica gel column chromatography and converted to their HCl salts by passing gaseous HCl through their solution in diethyl ether, which yielded the compounds in solid form. In the synthesis of oxime ether derivatives, the yield of derivatives with alkyl groups as side chains was particularly poor, but the yield of derivatives with aromatic rings and electronegative substituents was high. In the case of the 2,4-dichlorobenzyl-substituted derivative (**5k**), the reaction took less time (2 h), with a 60.28% yield due to the effect of both the aromatic ring and the electron-drawing chlorine substituents on the ring.

Structures of **5a**–**k** were confirmed using ^1^H-NMR and ^13^C-NMR, and mass spectrometry data. The signal for the oxime N-OH proton, which is usually observed around 12 ppm, was not detected in the ^1^H-NMR spectra of the title compounds; instead, alkyl protons for the oxime ether moiety were observed, which indicates that the oxime hydrogen was substituted by the alkyl groups. The protons on the carbon bound to the oxime oxygen were observed at about 4.14–4.80 ppm, and the arylalkyl (CH_2_-Ar) protons at the same position were observed as singlets in the range of 5.34–5.46 ppm. Other protons in the alkyl groups were found in places compatible with the NMR literature data.

### 2.2. Antimicrobial Activity

The title compounds were first tested against three different bacterial species: *Escherichia coli* (PeruMycA 3), *Bacillus subtilis* (PeruMycA 6), and *Staphylococcus aureus* (ATCC 6538). Except for **5i** and **5k**, they were all shown to be quite effective against *E. coli*. The presence of a methyl group in the R_1_ position resulted in better antimicrobial activity than trifluoromethyl against *E. coli*. The best MICs against *Escherichia coli* and *Bacillus subtilis*, on the other hand, were obtained with trifluoromethyl and isopropyl substituents at R_1_ and R_2_ positions (**5c**), respectively. This trend was not seen with *Staphylococcus aureus*. Compounds with 2-butenyl and *p*-chlorobenzyl groups as a tail showed reasonable activity against all the tested bacteria (Table 1). Collectively, they were more potent than the reference drug ciprofloxacin.

The effectiveness of the compounds against three different *Candida species* was also assessed, but they were shown to be less effective when compared to the standard drug fluconazole (Table 2).

However, when the compounds were tested against three dermatophytes, significantly low MIC values were observed when compared to the positive control griseofulvin. **5c** and **5h** showed remarkable activity, with MIC values as low as 1 μg/mL. Furthermore, **5g** and **5j** were quite effective, with MIC values lower than those of griseofulvin (Table 3). The antifungal activity results revealed that methyl and trifluoromethyl groups, R_1_, as well as isopropyl and *p*-chlorobenzyl groups, R_2_, led to substantial activity against *Trichophyton mentagrophytes* and *Arthroderma quadrifidum* species.

Overall, these newly synthesized compounds demonstrated both antifungal and antibacterial action. When the activity results against all bacterial and fungal strains were evaluated in general, no significant activity was observed in compounds with straight-chain aliphatic groups, R_2_ (tail), whereas activity increased with branched isopropyl groups. This finding was also supported by molecular modelling studies, which suggested that new compounds can be discovered by experimenting with branching isopropyl groups or groups with a near-electrical environment and volume. The effectiveness of the isopropyl derivatives was particularly pronounced in the case of 4-trifluoromethylphenyl derivative (**5c**); therefore, electron-withdrawing substituents (R_1_) could be preferred over electron-releasing substituents (R_1_) like methyl in the aryl group in future studies. Derivatives with an aromatic ring in the tail had relatively high activity, which, however, decreased by the introduction of an additional chlorine to the ring, as in **5j** and **5k**. As a result, **5c** emerged as a potential lead molecule with dual antifungal and antibacterial effects.

### 2.3. Molecular Modelling

Given the promising results obtained by this chemical scaffold towards bacterial and fungal species, we aimed at validating, in silico, some putative targets of pharmaceutical interest, namely lanosterol 14α-demethylase (CYP51) for fungal species and FtsZ (Filamenting temperature-sensitive mutant Z) protein for bacterial species.

**Figure 2:**
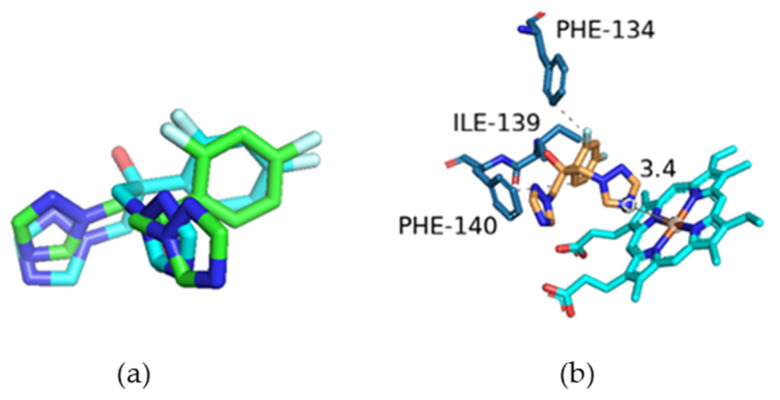
(**a**) Superimposition and (**b**) binding interaction of redocked ligand.

The redocking validation protocol of fluconazole (ligand) provided an RMSD value of 1.451 Å, which indicates the scoring function’s ability to predict crystal conformation. The superimposition of the redocked fluconazole and crystal ligand with their respective interactions with amino acids is given in Figure 2.

Oxime analogues can play an important role in maximizing potential interactions [28]. Briefly, **5c** has an oxime endowed with a propyl chain, and its benzene ring is substituted with the trifluoro (CF_3_) group, which can change the dipole of the structure due to its electronegative character. Furthermore, **5h** is characterized by an oxime with chlorobenzyl and 4-methylpenyl. Binding energies and interactions of these compounds were generated for further analysis and are represented in Table 4.

**Figure 3:**
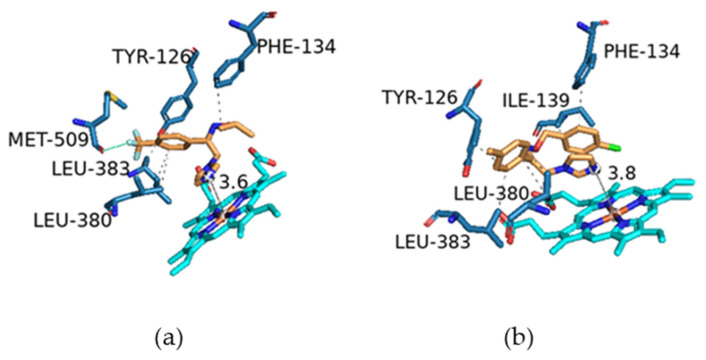
Binding interaction of **5c** (**a**) and **5h** (**b**) in the active site of CYP51.

Another important interaction is the interaction between the azole nitrogen and the heme cofactor. The conformation of a particular structure can affect the distance of the coordination bond between the iron (Fe) of heme and nitrogen of azole. Briefly, **5c** and **5h** had coordination bonds of 3.6 and 3.8 Å, respectively, while fluconazole had a distance of about 3.4 Å. The interaction of **5c** and **5h** with amino acid residues is given in Figure 3. The docking studies documented that compounds **5c** and **5h** comparatively displayed better binding energy than the standard FDA-approved fluconazole drug. The imidazole-derived lead molecules in the present study could interact with the cytochrome P-450 enzyme lanosterol 14α-demethylase, which catalyzes the pivotal conversion of lanosterol into ergosterol.

**Figure 4:**
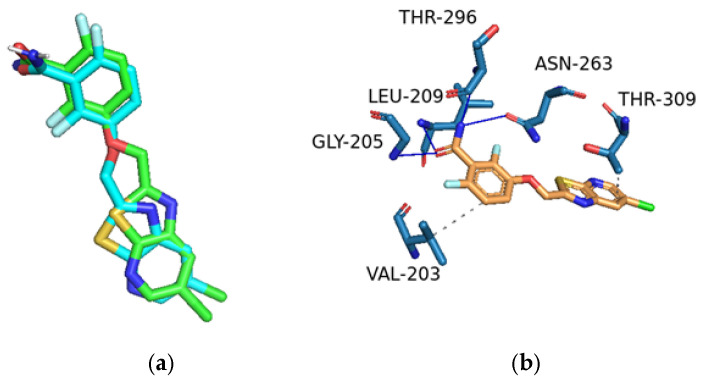
(**a**) Superimposition and (**b**) binding interaction of redocked ligand.

**Figure 5:**
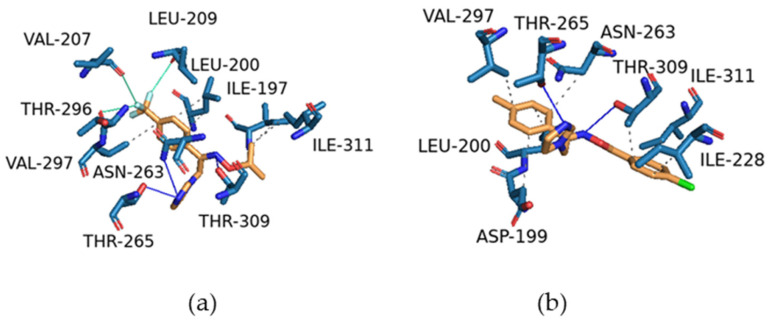
Binding interaction of **5c** (**a**) and **5h** (**b**) in the active site of protein FtsZ.

Successively, the redocking validation protocol of 9PC (ligand) gave an RMSD of 1.101 Å with a binding affinity of −9.7 kcal/mol, which indicates the scoring function’s ability to predict crystal conformation. The superimposition of the redocked standard and crystal ligand with its respective interactions with amino acids is given in Figure 4. Compound **5h** showed an affinity of −9.5 kcal/mol, comparable to the standard (−9.7 kcal/mol), even though there were only similar hydrophobic interactions to the standard, but it formed two H-bonds with THR 309 and THR 265, which might have contributed to its comparatively greater affinity than that of **5c**. Even though **5c** had lower affinity, there were several interesting interactions with the trifluoro (CF_3_) group of **5c**. This derivative formed halogen bonds with LEU 209 and THR 296, which also established hydrogen bonds with the standard. Additionally, hydrogen bonding with ASN 263 was seen in both the standard and compound **5c**. The binding energy and interactions of these compounds were generated for further analysis, as represented in Table 5; a 3D diagram of binding interactions is given in Figure 5.

## 3. Materials and Methods

### 3.1. Chemistry

All reagents and solvents were obtained from commercial suppliers and were of analytic purity. All reactions were monitored by analytical thin-layer chromatography (TLC) on silica-gel-precoated F_254_ Merck plates (Rahway, NJ, USA), which were examined under UV light at 254 nm. Melting points (mp) were recorded by an Electrothermal Digital Melting Point Apparatus (Cole-Parmer, Staffordshire, UK) without correction. ^1^H-NMR (300 MHz) and ^13^C-NMR (75 MHz) spectra were recorded with a Bruker Avonce 600 Ultrashield™ (Bremen, Germany) NMR spectrometer. Compounds were dissolved in dimethyl sulfoxide (DMSO-*d*_6_), and the chemical shifts are reported as δ (ppm) values using tetramethylsilane as an internal reference. The splitting patterns were noted as s (singlet), d (doublet), t (triplet), q (quartet), m (multiplet), and dd (doublet of doublet). The HRMS spectra of the synthesized compounds were obtained from their solutions in methanol using positive-ion (ESI+) electrospray ionization techniques with a Waters LCT Premier XE UPLC/MS TOFF system (Milford, MA, USA) and MassLynx 4.1 software (Supporting Information).

#### Preparation of the Azole Compounds

*2-Bromo-1-(4-trifluormethylphenyl)/1-(4-methylphenyl)ethanone (***2***)*

50 mmol 4-methylacetophenone in 50 mL acetic acid was stirred in an ice bath, to which the first 3 drops of HBr and then 50 mmol Br_2_ solution in 2.5 mL acetic acid were added dropwise with constant stirring at 0–5 °C. After bromine addition was complete, the reaction was stirred at room temperature for 2 h. The reaction medium was poured into ice water, and the precipitate was filtered, washed with sodium bicarbonate solution (7.5%), and dried in dark. It was purified by crystallization from a mixture of methanol/water [23].

*1-(4-trifluoromethylphenyl)/1-(4-methylphenyl)-2-(1H-imidazol-1-yl)ethanone (***3***)*

To a solution of 30 mmol imidazole in 2.5 mL DMF in an ice bath,10 mmol 2-bromo-1-(4-trifluoromethylphenyl)/1-(4-methylphenyl)ethanone in 2.5 mL DMF was slowly added by vigorously stirring the mixture at 0–5 °C. The reaction mixture was stirred for 2 h in an ice bath, then overnight at room temperature. The reaction medium was poured into ice water, and the precipitate was filtered and purified by crystallization from the methanol and water mixture [23].

*1-(4-Trifluoromethylphenyl)/1-(4-methylphenyl)-2-(1H-imidazol-1-yl)ethanone oxime (***4***)*

15 Mmol 1-(4-trifluoromethylphenyl)/1-(4-methylphenyl)-2-(1*H*-imidazol-1-yl)ethanone and 30 mmol hydroxylamine hydrochloride were dissolved in 75 mL ethanol and alkylated to pH = 14 with 15 N sodium hydroxide solution. The mixture was refluxed for 3 h, then poured into distillated water and acidified with concentrated hydrochloric acid to pH = 5. The resulting precipitate was filtered, washed with 5% sodium bicarbonate solution and dried [27].

*1-(4-Trifluoromethylphenyl)/1-(4-methylphenyl)-2-(1H-imidazol-1-yl)ethanone oxime ethers (***5a**–**k***)*

Briefly, 0.01 Mol 1-(4-trifluoromethylphenyl)/1-(4-methylphenyl)-2-(1*H*-imidazol-1-yl)ethanone oxime and 0.011 mol sodium ethoxide were stirred and refluxed for 30 min. Ethanol was evaporated in vacuo; the residue was dissolved in DMF, and 0.02 mol of the appropriate alkyl halide was added. The mixture was stirred at room temperature for 4 h and then poured onto ice. The mixture was extracted with chloroform, and the organic layer was separated and evaporated to dryness. The oily residue that remained was not solidified, prompting the purification of the compounds through column chromatography utilizing HPLC-grade methanol and chloroform as mobile phases in a 95 to 5 ratio. The eluents collected in the test tubes were checked by thin-layer chromatography and evaporated. The residue was dissolved in the ether with gaseous HCl to give a solid [27]. All spectral data of the compounds were in accordance with the assigned structures, as presented below.

*1-(4-Methylphenyl)-2-(1H-imidazol-1-yl)ethanone O-methyloxime hydrochloride (***5a***)*

The general procedure was followed using 1-(4-methylphenyl)-2-(1*H*-imidazol-1-yl)ethanone oxime and 1-iodomethane to give **5a** as a white solid (yield 16.15%, m.p. 168 °C). ^1^H-NMR (CDCl_3_, 300 MHz) δ 2.32 (s, 3H, phenyl-CH_3_), 4.01 (s, 3H, O-CH_3_), 5.56 (s, 2H, CH_2_N), 7.21–7.97 (m, 7H, phenyl and imidazole), 9.16 (s, 1H, HCl). ^13^C-NMR (DMSO-*d*_6_, 75 MHz), 21.2 (1C; CH_3_), 42.8 (1C; CH_3_-O), 62.9 (1C; CH_2_-N), 128.7, 129.4, 129.7, 129.8, 130.1 (6C, phenyl), 136.6, 139.6 (2C; imidazole C^4,5^), 140.2 (1C; imidazole C^2^), 151.8 (1C; C=N). C_13_H_16_ClN_3_O MS (ESI+) (Calc.: 230.1293; Found: 230.1290) ([M + H]^+^ 100.0%).

*1-(4-Methylphenyl)-2-(1H-imidazol-1-yl)ethanone O-ethyloxime hydrochloride (***5b***)*

The general procedure was followed using 1-(4-methylphenyl)-2-(1*H*-imidazol-1-yl)ethanone oxime and 1-bromoethane to give **5b** as a white solid (yield 38.07%, m.p. 142 °C). ^1^H-NMR (CDCl_3_, 300 MHz) δ 1.28 (t, 3H, CH_3_CH_2_-), 2.32 (s, 3H, phenyl-CH_3_), 4.26 (q, 2H, CH_3_CH_2_-), 5.59 (s, 2H, CH_2_N), 7.22–7.69 (m, 7H, phenyl and imidazole), 9.25 (s, 1H, HCl). ^13^C-NMR (DMSO-*d*_6_, 75 MHz), 14.8 (1C; CH_3_), 21.3 (1C; CH_3_), 43.1 (1C; CH_2_-O), 70.6 (1C; CH_2_-N), 122.7, 126.9, 128.7, 129.8, 130.1 (6C, phenyl), 136.5, 139.6 (2C; imidazole C^4,5^), 140.2 (1C; imidazole C2), 151.5 (1C; C=N). C_14_H_18_ClN_3_O MS (ESI+) (Calc.: 244.1450; Found: 244.1443) ([M + H]^+^ 100.0%).

*1-(4-Trifluoromethylphenyl)-2-(1H-imidazol-1-yl)ethanone O-isopropyloxime hydrochloride (***5c***)*

The general procedure was followed using 1-(4-trifluoromethylphenyl)-2-(1*H*-imidazol-1-yl)ethanone oxime and 2-bromopropan to give **5c** as a white solid (yield 32.15%, m.p. 134 °C). ^1^H-NMR (CDCl_3_, 300 MHz) δ 1.26–1.28 (d, 6H, (CH_3_)_2_CH-, j:3), 4.48–4.52 (m, 1H, CH), 5.63 (s, 2H, CH_2_N), 7.62–7.98 (m, 7H, phenyl and imidazole), 9.28 (s, 1H, HCl). ^13^C-NMR (DMSO-*d*_6_, 75 MHz) 21.6, 21.8 (2C; (CH_3_)_2_CH-), 52.3 (1C; CH-O), 77.6 (1C; CH_2_-N), 120.3 (1C; CF_3_), 121.6, 122.8, 123.6, 125.4, 126.0, 127.9 (6C, phenyl), 130.4, 130.6 (2C; imidazole C^4,5^), 136.6 (1C; imidazole C^2^), 150.2 (1C; C=N). C_15_H_17_ClF_3_N_3_O MS (ESI+) (Calc.: 312.1324; Found: 312.1312) ([M + H]^+^ 100.0%).

*1-(4-Methylphenyl)-2-(1H-imidazol-1-yl)ethanone O-isopropyloxime hydrochloride (***5d***)*

The general procedure was followed using 1-(4-methylphenyl)-2-(1*H*-imidazol-1-yl)ethanone oxime and 2-bromopropane to give **5d** as a white solid (yield 59.53%, m.p. 160 °C). ^1^H-NMR (CDCl_3_, 300 MHz) δ 1.26–1.27 (d, 6H, (CH_3_)_2_CH-, j:3), 2.31 (s, 3H, phenyl-CH_3_) 4.45–4.49 (m, 1H, CH), 5.56 (s, 2H, CH_2_N), 7.22–7.64 (m, 7H, phenyl and imidazole), 9.25 (s, 1H, HCl). ^13^C-NMR (DMSO-*d*_6_, 75 MHz) 21.3, 21.9 (2C; (CH_3_)_2_CH-), 43.2 (1C; CH-O), 76.9 (1C; CH_2_-N), 120.5, 122.7, 126.9, 129.8, (6C, phenyl), 130.4, 136.2 (2C; imidazole C^4,5^), 140.1 (1C; imidazole C^2^), 150.9 (1C; C=N). C_15_H_20_ClN_3_O MS (ESI+) (Calc.: 258.1606; Found: 258.1602) ([M + H]^+^ 100.0%).

*1-(4-Methylphenyl)-2-(1H-imidazol-1-yl)ethanone O-hexyloxime hydrochloride (***5e***)*

The general procedure was followed using 1-(4-methylphenyl)-2-(1*H*-imidazol-1-yl)ethanone oxime and 1-bromhexane to give **5e** as a white solid (yield 40.14%, m.p. 182 °C). ^1^H-NMR (CDCl_3_, 300 MHz) δ 0.86 (t, 3H, CH_3_CH_2_-), 1.21–1.32 (m, 6H, CH_3_CH_2_CH_2_CH_2_-), 1.63–1.68 (m, 2H, -CH_2_-), 2.31 (s, 3H, phenyl-CH_3_), 4.23 (t, 2H, -CH_2_-O), 5.57 (s, 2H, -CH_2_N), 7.22–7.71 (m, 7H, phenyl and imidazole), 9.23 (s, 1H, HCl). ^13^C-NMR (DMSO-*d*_6_, 75 MHz), 14.3 (1C; CH_3_), 21.3 (1C; CH_2_), 22.5 (1C; CH_2_), 25.4 (1C; CH_2_), 29.0 (1C; CH_2_), 34.5 (s, 3H, phenyl-CH_3_), 43.2 (1C; CH_2_-O), 75.1 (1C; CH_2_-N), 120.5, 122.7, 126.9, 128.7, 129.4, 129.8 (6C, phenyl), 130.2, 136.5 (2C; imidazole C^4,5^), 140.2 (1C; imidazole C^2^), 151.2 (1C; C=N). C_18_H_26_ClN_3_O MS (ESI+) (Calc.: 300.2000; Found: 300.2062) ([M + H]^+^ 100.0%).

*1-(4-Methylphenyl)-2-(1H-imidazol-1-yl)ethanone O-crotyloxime hydrochloride (***5f***)*

The general procedure was followed using 1-(4-methylphenyl)-2-(1*H*-imidazol-1-yl)ethanone oxime and crotyl chloride to give **5f** as a white solid (yield 42.35%, m.p. 108 °C). ^1^H-NMR (CDCl_3_, 300 MHz) δ 1.69–1.70 (d, 3H, CH_3_CH=, j:3), 2.30 (s, 3H, phenyl-CH_3_), 4.66–4.67 (d, 2H, OCH_2_-CH), 5.59 (s, 2H, CH_2_N), 5.67–5.69 (d of d, 1H, -CH=CH-, j:6), 5.67–5.68, 5.71–5.75(d of d, 1H, -CH=CH-, j:6), 5.78–5.79, 5.81–5.82 (d of d, 1H, -CH=CH-, j:3), 7.21–7.75 (m, 7H, phenyl and imidazole), 9.25 (s, 1H, HCl). ^13^C-NMR (DMSO-*d*_6_, 75 MHz), 13.7 (1C; CH_3_), 21.3 (1C; CH_3_), 43.2 (1C; CH_2_-O), 75.6 (1C; CH_2_-N), 120.5, 122.7, 127.0, 128.7, 129.8, 130.6 (6C, phenyl), 130.9, 136.5 (2C; imidazole C^4,5^), 140.2 (1C; imidazole C^2^), 151.7 (1C; C=N), 151.8, 162.8 (2C, CH=CH). C_16_H_20_ClN_3_O MS (ESI+) (Calc.: 270.1606; Found: 270.1600) ([M + H]^+^ 100.0%).

*1-(4-Methylphenyl)-2-(1H-imidazol-1-yl)ethanone O-cyclohexylmethyloxime hydrochloride (***5g***)*

The general procedure was followed using 1-(4-methylphenyl)-2-(1*H*-imidazol-1-yl)ethanone oxime and cyclohexyl methyl chloride to give **5g** as a white solid (yield 22.51%, m.p. 198 °C). ^1^H-NMR (CDCl_3_, 300 MHz) δ 0.91–1.73 (m, 11H, cyclohexyl protons), 2.31 (s, 3H, phenyl-CH_3_), 4.03–4.04 (d, 2H, CH_2_-O, j:3), 5.58 (s, 2H, CH_2_N), 7.22–7.65 (m, 7H, phenyl and imidazole), 9.24 (s, 1H, HCl). ^13^C-NMR (DMSO-*d*_6_, 75 MHz) 21.3 (1C; CH_3_), 25.7, 26.5, 29.5, 37.5 (6C; cyclohexyl carbons), 43.3 (1C; CH_2_-O), 80.4 (1C; CH_2_-N), 120.5, 122.7, 126.9, 129.8, 130.2 (6C, phenyl), 136.5 (2C; imidazole C^4,5^), 140.2 (1C; imidazole C^2^), 151.1 (1C; C=N). C_19_H_26_ClN_3_O MS (ESI+) (Calc.: 312.2076; Found: 312.2068) ([M + H]^+^ 100.0%).

*1-(4-Methylphenyl)-2-(1H-imidazol-1-yl)ethanone O-(4-chlorophenyl)methyloxime hydrochloride (***5h***)*

The general procedure was followed using 1-(4-methylphenyl)-2-(1*H*-imidazol-1-yl)ethanone oxime and 1-bromoethan to give **5h** as a white solid (yield 36.80%, m.p. 169 °C). ^1^H-NMR (CDCl_3_, 300 MHz) δ 2.36 (s, 3H, phenyl-CH_3_), 5.27 (s, 2H, phenyl-CH_2_-O), 5.66 (s, 2H, CH_2_N), 6.99–7.66 (m, 11H, phenyl and imidazole), 9.66 (s, 1H, HCl). ^13^C-NMR (DMSO-*d*_6_, 75 MHz) 21.3 (1C; CH_3_), 42.4 (1C; CH_2_-O), 76.7 (1C; CH_2_-N), 119.9, 120.7, 126.3, 128.8, 128.9, 129.9, 130.1, 134.5 (12C, phenyl), 134.9, 135.9 (2C; imidazole C^4,5^), 141.2 (1C; imidazole C^2^), 150.7 (1C; C=N). C_19_H_19_C_l2_N_3_O MS (ESI+) (Calc.: 340.1217; Found: 340.1220) ([M + H]^+^ 100.0%).

*1-(4-Trifluoromethylphenyl)-2-(1H-imidazol-1-yl)ethanone O-(4-chlorophenyl)methyloxime hydrochloride (***5i***)*

The general procedure was followed using 1-(4-methylphenyl)-2-(1*H*-imidazol-1-yl)ethanone oxime and 1-bromoethan to give **5i** as a white solid (yield 38.88%, m.p. 89 °C). ^1^H-NMR (CDCl_3_, 75 MHz) δ 5.34 (s, 2H, phenyl-CH_2_-O), 5.43 (s, 2H, CH_2_N), 6.81–7.86 (m, 11H, phenyl and imidazole). ^13^C-NMR (DMSO-*d*_6_, 300 MHz) 49.4 (1C; CH_2_-O), 75.9 (1C; CH_2_-N), 119.9, 119.9 (1C; CF_3_), 123.5, 125.3, 127.8, 128.9, 128.9, 129.1, 129.3, 129.4, 130.1, 130.4, 130.6 (11C, phenyl), 133.0, 133.2 (1C; =CH-CF_3_), 136.7, 137.3 (2C; imidazole C^4,5^), 138.2 (1C; imidazole C^2^), 153.4 (1C; C=N). C_19_H_15_ClF_3_N_3_O MS (ESI+) (Calc.: 394.0934; Found: 394.0929) ([M + H]^+^ 100.0%).

*1-(4-Methylphenyl)-2-(1H-imidazol-1-yl)ethanone O-(2,4-dichlorophenyl)methyloxime hydrochloride (***5j***)*

The general procedure was followed using 1-(4-methylphenyl)-2-(1*H*-imidazol-1-yl)ethanone oxime and 1-bromoethan to give **5j** as a white solid (yield 36.79%, m.p. 136 °C). ^1^H-NMR (CDCl_3_, 300 MHz) δ 2.36 (s, 3H, CH_3_), 5.27 (s, 2H, phenyl-CH_2_-O), 5.66 (s, 2H, CH_2_N), 7.22–7.68 (m, 10H, phenyl and imidazole), 9.23 (s, 1H, HCl). ^13^C-NMR (DMSO-*d*_6_, 75 MHz) 21.3 (1C; CH_3_), 43.2 (1C; CH_2_-O), 73.4 (1C; CH_2_-N), 120.6, 122.7, 127.1, 127.9, 129.4, 129.7, 129.8, 132.5, 134.1, 134.2, 134.2 (12C, phenyl), 136.6 (2C; imidazole C^4,5^), 140.6 (1C; imidazole C^2^), 152.8 (1C; C=N). C_19_H_18_C_l3_N_3_O MS (ESI+) (Calc.: 374.0827; Found: 374.0838) ([M + H]^+^ 100.0%).

*1-(4-Trifluoromethylphenyl)-2-(1H-imidazol-1-yl)ethanone O-(2,4-dichlorophenyl)methyloxime hydrochloride (***5k***)*

The general procedure was followed using 1-(4-methylphenyl)-2-(1*H*-imidazol-1-yl)ethanone oxime and 1-bromoethan to give **5k** as a white solid (yield 60.28%, m.p. 116 °C). ^1^H-NMR (CDCl_3_, 300 MHz) δ 5.27 (s, 2H, phenyl-CH_2_-O), 5.66 (s, 2H, CH_2_N), 6.85–7.72 (m, 10H, phenyl and imidazole). ^13^C-NMR (DMSO-*d*_6_, 75 MHz) 40.6 (1C; CH2-O), 74.0 (1C; CH2-N), 119.2 (1C; CF_3_), 125.8, 126.6, 127.3, 128.0, 129.6, 129.8, 131.4, 131.8, 132.1, 132.9, 134.8 (11C, phenyl), 132.1, 132.9 (1C; =CH-CF_3_), 135.1, 136.6 (2C; imidazole C^4,5^), 137.5 (1C; imidazole C^2^), 151.7 (1C; C=N). C_19_H_14_Cl_2_F_3_N_3_O MS (ESI+) (Calc.: 428.0544; Found: 428.0539) ([M + H]^+^ 100.0%).

### 3.2. Antimicrobial Assays

The in vitro antimicrobial activity of the newly synthesized compounds was assessed against three Gram-negative and Gram-positive bacterial species, namely *B. subtilis* (PeruMycA 6), *S. aureus* (ATCC 6538), and *E. coli* (PeruMycA 3); three yeasts from *Candida* genus, namely *C. tropicalis* (YEPGA 6184), *C. albicans* (YEPGA 6379), and *C. parapsilosis* (YEPGA6551); and three dermatophytes, namely *T. mentagrophytes* (CCF 4823), *A. quadrifidum* (CCF 5792), and *A. gypseum* (CCF 6261). The tested fungal and bacterial strains were from the ATCC (from https://www.atcc.org/), CCF (Culture Collection of Fungi, from the Department of Botany, Faculty of Science, Charles University in Prague, Prague, Czech Republic), and PeruMycA (from the Department of Chemistry, Biology and Biotechnology, University of Perugia, Perugia, Italy) cultures, and are available upon request. The anti-microbial activities were compared to those of the reference drugs ciprofloxacin (CIP), fluconazole (FLU), and griseofulvin (GRI) for antibacterial, antifungal, and antidermatophytal activities, respectively. Tested compounds were prepared as 3 mg/mL stock solutions in dimethylsulphoxide (DMSO) and then used in the range 0.195–200 µg/mL. Each experiment of minimum inhibitory concentration (MIC) evaluation was performed in triplicate. Geometric means and MIC ranges were calculated. MICs on bacterial strains were determined according to the broth microdilution method of the Clinical and Laboratory Standards Institute (CLSI) [29]. Susceptibility testing against yeasts and filamentous fungi was performed according to the CLSI protocols [30,31,32]. The experimental conditions have already been reported [33].

### 3.3. Molecular Modelling Studies

Molecular docking of the present study was carried out using MzDOCK v. 2.9 software, which is a GUI-based open-source software containing an effective pipeline for the docking process [34]. Protein PDB ID: 4ZDZ [15] was chosen for this particular study, which contains a well-known antifungal standard, fluconazole (resname: TPF), in the ligand-binding cavity of the enzyme. The protein was prepared by removing water molecules and HET groups, as well as adding hydrogen and Kollman charges [35,36]. Cofactor heme (resname: HEM), which plays an important role in interaction in the standard, was retained with the protein preparation wizard of MzDOCK during the docking simulation. Compounds **5c** and **5h** were drawn using the JSME editor integrated with MzDOCK, and the drawn structures were prepared, modelled and energy-optimized with force field MMFF94 with a protonation state of 7.4 pH [37,38]. The ligand-binding site was configured at the position of the co-crystallized ligand fluconazole (TPF), and the search space was set to 4 Å from the co-crystallized ligand. The number of modes was set to 50 to enhance proper sampling for docking. MzDOCK utilizes docking engine Smina, which is a fork of Autodock Vina 1.2.5 [39,40]. Docking was run and the results, with an analysis image of interactions, were generated. For validation of docking, fluconazole was redocked and the RMSD between the crystal ligand and docked ligand was calculated with DockRMSD [41]. For studying the antibacterial properties of the lead molecules, it is possible to calculate the binding affinity and interactions with the FtsZ protein PDB ID: 4DXD [42]. All parameters used for docking in the target for studying the previously mentioned antifungal target were maintained in this docking approach. The co-crystallized ligand (resname: 9PC; 3-[(6-chloro[1,3]thiazolo[5,4-*b*]pyridin-2-yl)methoxy]-2,6-difluorobenzamide) displayed IC_50_ 150–153 nM, acting as a standard for comparisons of affinity as well as of binding interactions for lead compounds. No co-factors were involved in affecting the binding conformation; hence, no heteroatoms were retained.

## 4. Conclusions

In search of a hit for eradicating microbial infections, we synthesized a series of oxiconazole analogues and investigated them against a variety of bacteria and fungi. We acquired very powerful compounds with low MIC values against fungal dermatophytes, surpassing those of griseofulvin, the current standard for antifungal therapy. The title compounds also displayed highly potent activity against the tested bacteria, and the compounds **5c** and **5h** stood out with their dual antifungal and antibacterial effects. Molecular docking revealed that both compounds showed good binding energy with fungal CYP51 when compared with the native ligand. In case of the antibacterial target (FtsZ protein), the **5h** compound showed a comparable affinity of −9.5 kcal/mol to the standard −9.7 kcal/mol. The presence of more hydrophobic interactions with the lead molecules could produce excellent binding energies. This study provides a series of imidazole-based oxime ethers as a promising starting point to develop new agents combining antifungal and antibacterial efficacy in the fight against infectious diseases.

## Figures and Tables

**Table 1:** MIC values of **5a**–**k** against three bacterial strains.

Compounds	MIC (µg mL^−1^) *		
	*Escherichia coli* (PeruMycA 3)	*Bacillus subtilis* (PeruMycA 6)	*Staphylococcus aureus* (ATCC 6538)
**5a**	4.96 (±1.8)	4.96 (±1.8)	7.87 (±3.6)
**5b**	1.61 (±1.13)	2.47 (±0.90)	9.92 (±3.6)
**5c**	1.96 (±0.90)	1.23 (±1.35)	15.75 (±7.21)
**5d**	1.96 (±0.90)	3.93 (±1.8)	31.49 (±14.4)
**5e**	4.96 (±1.8)	3.93 (±1.8)	2.47 (±0.9)
**5f**	1.96 (±0.90)	2.47 (±0.9)	3.93 (±1.8)
**5g**	3.93 (±1.8)	4.96 (±1.8)	7.87 (±3.6)
**5h**	1.96 (±0.90)	1.96 (±0.90)	3.93 (±1.8)
**5i**	31.49 (±14.4)	7.87 (±3.6)	>200
**5j**	9.92 (±3.6)	3.93 (±1.8)	62.99 (±28.8)
**5k**	19.84 (±7.21)	15.75 (±7.21)	9.92 (±3.6)
**Ciprofloxacin**	31.49 (±14.4)	125.99 (±57.7)	200–>200

* MIC values are reported as mean (±SD) of three independent replicates (*n* = 3).

**Table 2:** MIC values of **5a**–**k** against three *Candida* species.

Compounds	MIC (µg mL^−1^) *		
	*Candida tropicalis* (YEPGA 6184)	*Candida albicans* (YEPGA 6379)	*Candida parapsilosis* (YEPGA 6551)
**5a**	62.99 (±28.86)	>200	31.49 (±14.43)
**5b**	7.87 (±3.6)	>200	62.99 (±28.86)
**5c**	125.99 (±57.33)	>200	39.68 (±14.43)
**5d**	79.37 (±28.86)	158.74 (±57.73)	62.99 (±28.86)
**5e**	31.49 (±14.43)	79.37 (±28.86)	31.49 (±14.43)
**5f**	158.74 (±57.73)	>200	62.99 (±28.86)
**5g**	79.37 (±28.86)	125.99 (±57.73)	31.49 (±14.43)
**5h**	158.74 (±57.73)	>200	79.37 (±28.86)
**5i**	>200	>200	79.37 (±28.86)
**5j**	125.99 (±57.33)	62.99 (±28.86)	39.68 (±14.43)
**5k**	>200	31.49 (±14.43)	62.99 (±28.86)
**Fluconazole**	1.81 (± 0.28)	1.25 (±0.57)	3.17 (±1.15)

* MIC values are reported as mean (±SD) of three independent replicates (*n* = 3).

**Table 3:** MIC values of **5a**–**k** against three dermatophytes.

Compounds	MIC (µg mL^−1^) *		
	*Trichophyton mentagrophytes* (CCF 4823)	*Arthroderma quadrifidum* (CCF 5792)	*Arthroderma gypseum* (CCF 6261)
**5a**	31.49 (±14.43)	15.74 (±7.21)	39.68 (±14.43)
**5b**	15.74 (±7.21)	7.87 (±7.21)	9.92 (±3.6)
**5c**	0.491 (±0.22)	0.619 (±0.22)	7.87 (±3.6)
**5d**	15.74 (±7.21)	2.48 (±0.90)	15.74 (±7.21)
**5e**	7.87 (±7.21)	2.48 (±0.90)	1.968 (±0.90)
**5f**	15.74 (±7.21)	7.87 (±7.21)	31.49 (±14.43)
**5g**	1.239 (±0.45)	3.937 (±1.8)	1.968 (±0.90)
**5h**	0.619 (±0.22)	0.983 (±0.45)	2.48 (±0.90)
**5i**	4.98 (±1.8)	2.48 (±0.90)	3.937 (±1.8)
**5j**	1.239 (±0.45)	1.968 (±0.90)	1.968 (±0.90)
**5k**	3.937 (±1.8)	1.23 (±0.45)	15.74 (±7.21)
**Griseofulvin**	2.52 (±1.15)	>8	3.174 (±1.15)

*MIC values are reported as mean (±SD) of three independent replicates (*n* = 3).

**Table 4:** Binding energy of lead molecules and reference standard (fluconazole) in the active site of CYP51, obtained by using MzDOCK.

Code	Binding Energy (Kcal)	Type of Interactions	Amino Acids Involved	H-DIST (Å)
**5c**	−8.1	Hydrophobic	TYR 126	3.65
		Hydrophobic	PHE 134	3.52
		Hydrophobic	LEU 380	3.94
		Hydrophobic	LEU 380	3.91
		Hydrophobic	LEU 383	3.65
		Halogen Bond	MET 509	3.03
**5h**	−8.2	Hydrophobic	TYR 126	3.57
		Hydrophobic	PHE 134	3.70
		Hydrophobic	ILE 139	3.79
		Hydrophobic	LEU 380	3.34
		Hydrophobic	LEU 380	3.84
		Hydrophobic	LEU 383	3.23
**Fluconazole**	−7.0	Hydrophobic	PHE 134	3.71
		Hydrophobic	ILE 139	3.76
		Hydrophobic	PHE 140	3.98

**Table 5:** Binding energy of lead molecules and reference standard (9PC) towards FtsZ, obtained by using MzDOCK.

Code	Binding Energy (Kcal)	Type of Interactions	Amino Acid Residues Involved	H-DIST (Å)
**5c**	−7.0	Hydrophobic	ILE 197	3.85
		Hydrophobic	LEU 200	3.52
		Hydrophobic	LEU 200	3.42
		Hydrophobic	ASN 263	3.55
		Hydrophobic	VAL 297	3.42
		Hydrophobic	ILE 311	3.74
		H-Bond	ASN 263	2.67
		H-Bond	THR 265	2.41
		H-Bond	THR 309	2.25
		Halogen Bond	VAL 207	3.72
		Halogen Bond	LEU 209	3.16
		Halogen Bond	THR 296	3.69
**5h**	−9.5	Hydrophobic	ASP 199	3.91
		Hydrophobic	LEU 200	3.66
		Hydrophobic	LEU 200	3.48
		Hydrophobic	ILE 228	3.80
		Hydrophobic	ASN 263	3.67
		Hydrophobic	VAL 297	3.39
		Hydrophobic	THR 309	3.40
		Hydrophobic	ILE 311	3.78
		H-Bond	THR 265	2.42
		H-Bond	THR 309	2.27
**9PC**	−9.7	Hydrophobic	VAL 203	3.99
		Hydrophobic	THR 309	3.68
		H-Bond	GLY 205	2.74
		H-Bond	LEU 209	1.99
		H-Bond	ASN 263	1.91
		H-Bond	THR 296	3.09

## Data Availability

This published article and its Supplementary Materials include all the data generated or analyzed during this study.

## Supplementary Materials

The following supporting information can be downloaded at https://www.mdpi.com/article/10.3390/antibiotics13111044/s1: ESI-MS spectra of the newly synthesized compounds.
