# Supplementary material for: Potent Antimicrobial Azoles: Synthesis, In Vitro and In Silico Study
Source: Antibiotics (Basel). 2024 Nov 4;13(11):1044. doi: 10.3390/antibiotics13111044 (PMC11591103; doi:10.3390/antibiotics13111044)
Supplement: Supplementary file 1 [file antibiotics-13-01044-s001.zip › antibiotics-3269284-supplementary.pdf]

Supplementary material

Potent Antimicrobial Azoles: Synthesis, in vitro and in silico study

Zeynep Özdemir[1], Yaren Nur Zenni[1], Arzu Karakurt[2], Suat Sari[3], Selma Saraç[4], Mevlüt Akdağ[5], İrem Bozbey Merde[1], Didem Kart[6], Roberto Venanzoni[7], Giancarlo Angeles Flores[7], Paola Angelini[7], Muzammil Kabier[8], Bijo Mathew[8*], Simone Carradori[9*]

[1]Department of Pharmaceutical Chemistry, Faculty of Pharmacy, Inonu University, Malatya, 44280, Türkiye
[2]Department of Pharmaceutical Chemistry, Faculty of Pharmacy, Lokman Hekim University, Ankara, 06100, Türkiye
[3]Department of Pharmaceutical Chemistry, Faculty of Pharmacy, Hacettepe University, Ankara, 06100, Türkiye.
[4]Department of Pharmaceutical Chemistry, Faculty of Pharmacy, Baskent University, Ankara, 06100, Türkiye
[5]Department of Pharmaceutical Chemistry, Faculty of Pharmacy, Afyonkarahisar Health Sciences University, Afyonkarahisar, 03030, Türkiye
[6]Department of Pharmaceutical Microbiology, Faculty of Pharmacy, Hacettepe University, Ankara, 06100, Türkiye
[7]Department of Chemistry, Biology and Biotechnology, University of Perugia, Perugia 06123, Italy
[8]Department of Pharmaceutical Chemistry, Amrita School of Pharmacy, Amrita Vishwa Vidyapeetham, AIMS Health Sciences Campus, Kochi, 682 041, India
[9]Department of Pharmacy, "G. d'Annunzio" University of Chieti-Pescara, Chieti 66100, Italy

***Correspond ng authors:** Simone Carradori PhD, Department of Pharmacy, "G. d'Annunzio" University of Chieti-Pescara, Chieti 66100, Italy; Tel. +39 0871 3554583; e-mail: simone.carradori@unich.it
Bijo Mathew, Department of Pharmaceutical Chemistry, Amrita School of Pharmacy, Amrita Vishwa Vidyapeetham, AIMS Health Sciences Campus, Kochi, 682 041, India; e-mail: bijovilaventgu@gmail.com; bijomathew@aims.amrita.edu

Experimental procedures

ESI-MS spectra of the newly synthesized compounds (pages 2-7)

HRMS spectrum of compound 5a

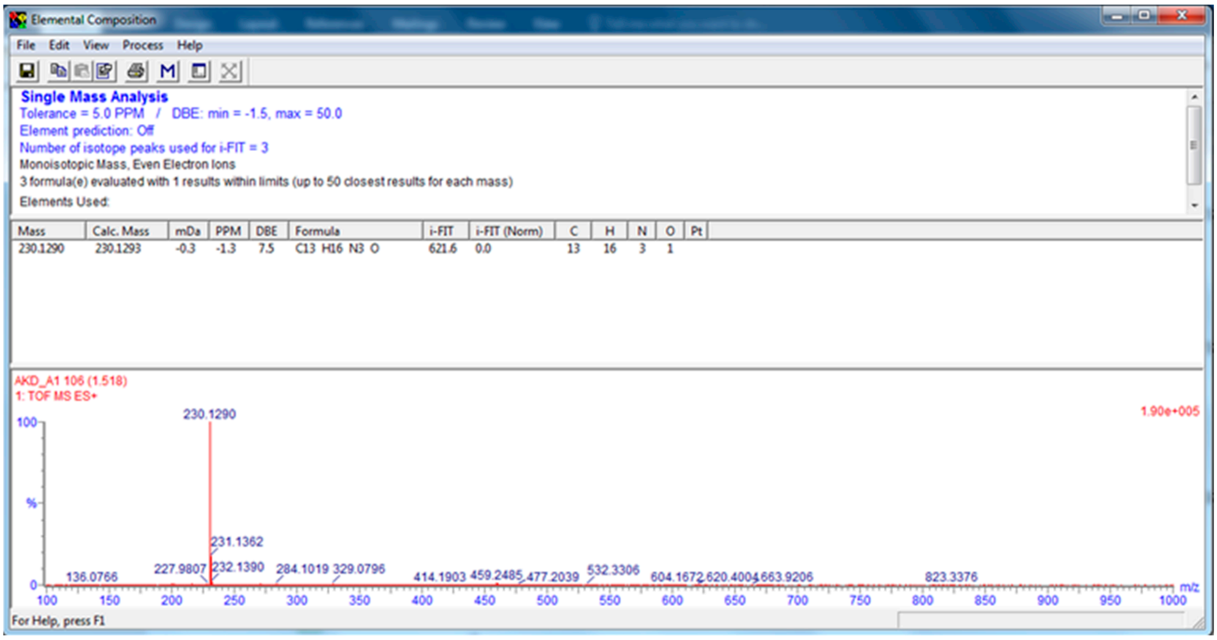

HRMS spectrum of compound 5b

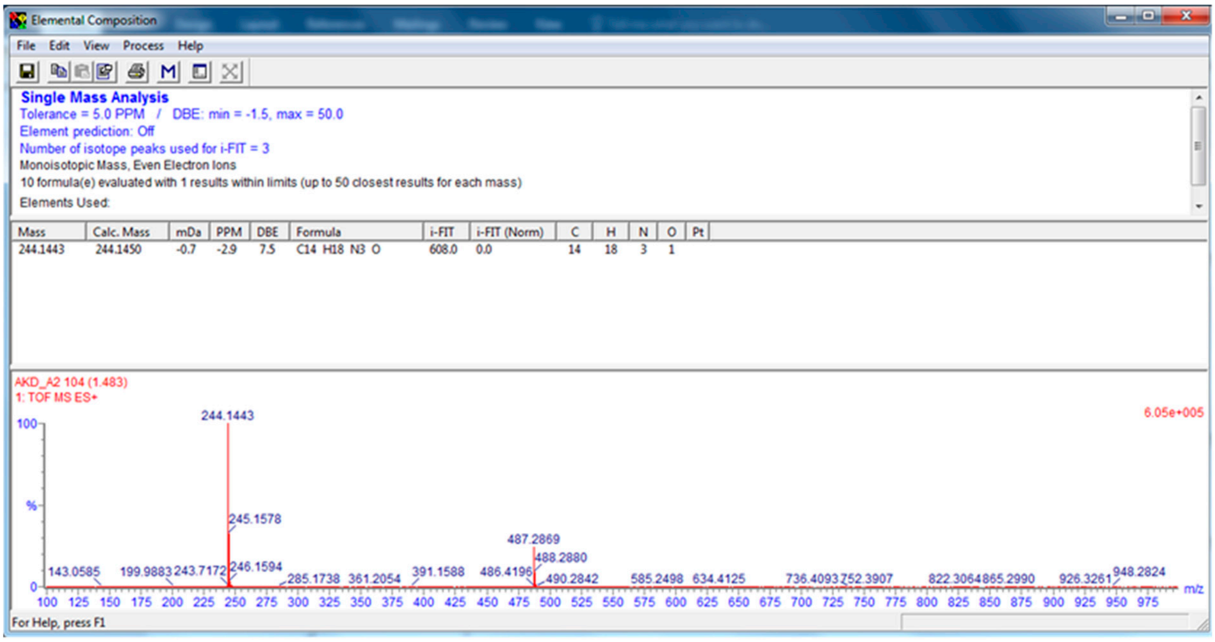

HRMS spectrum of compound 5c

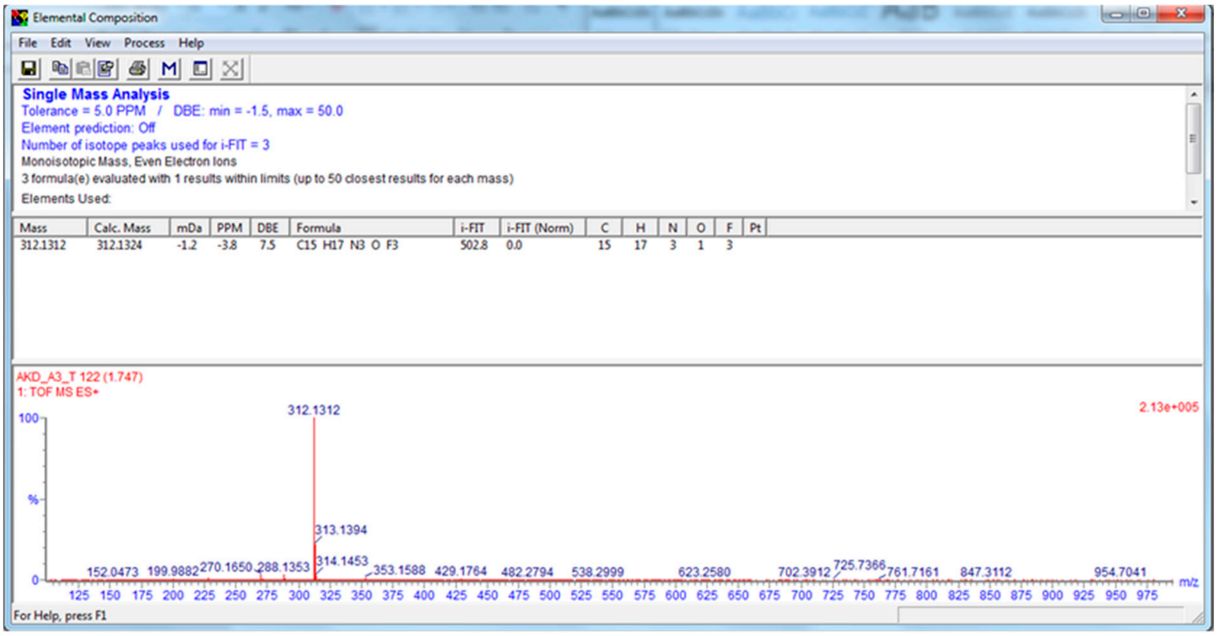

HRMS spectrum of compound 5d

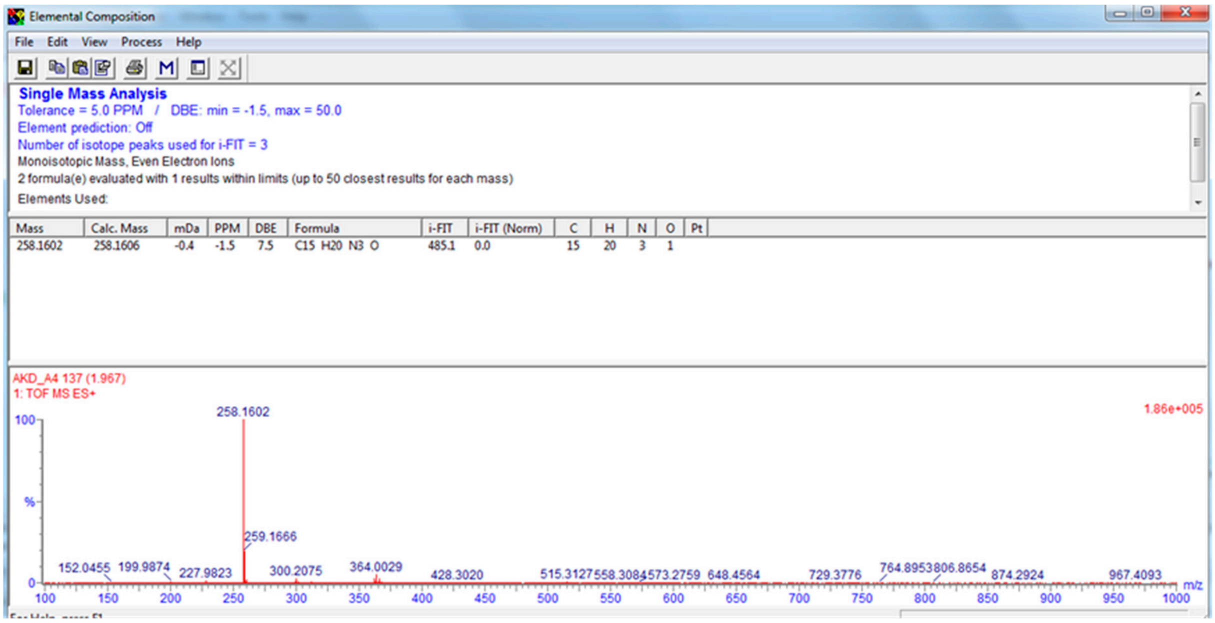

HRMS spectrum of compound 5e

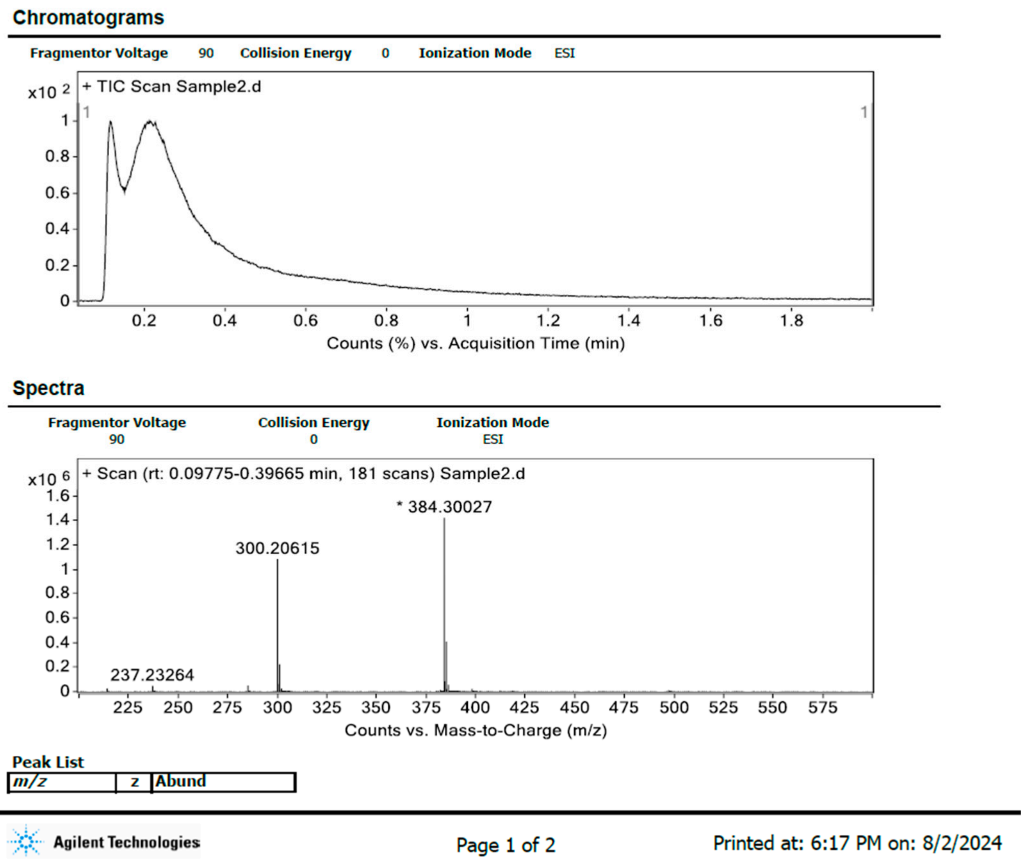

HRMS spectrum of compound 5f

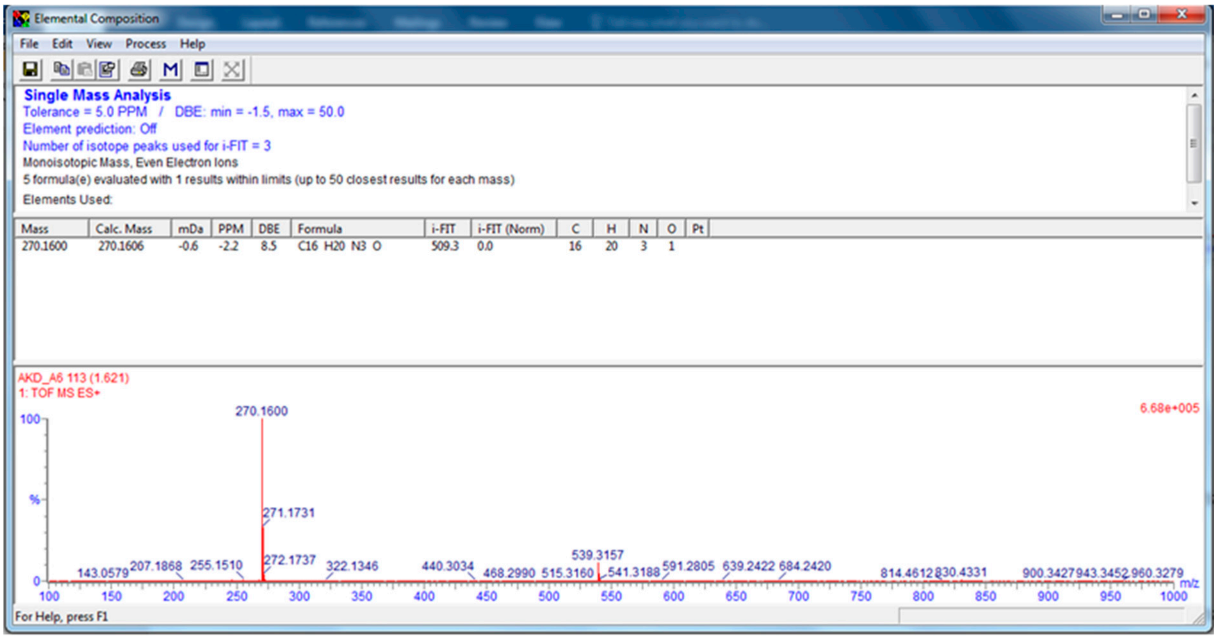

HRMS spectrum of compound 5g

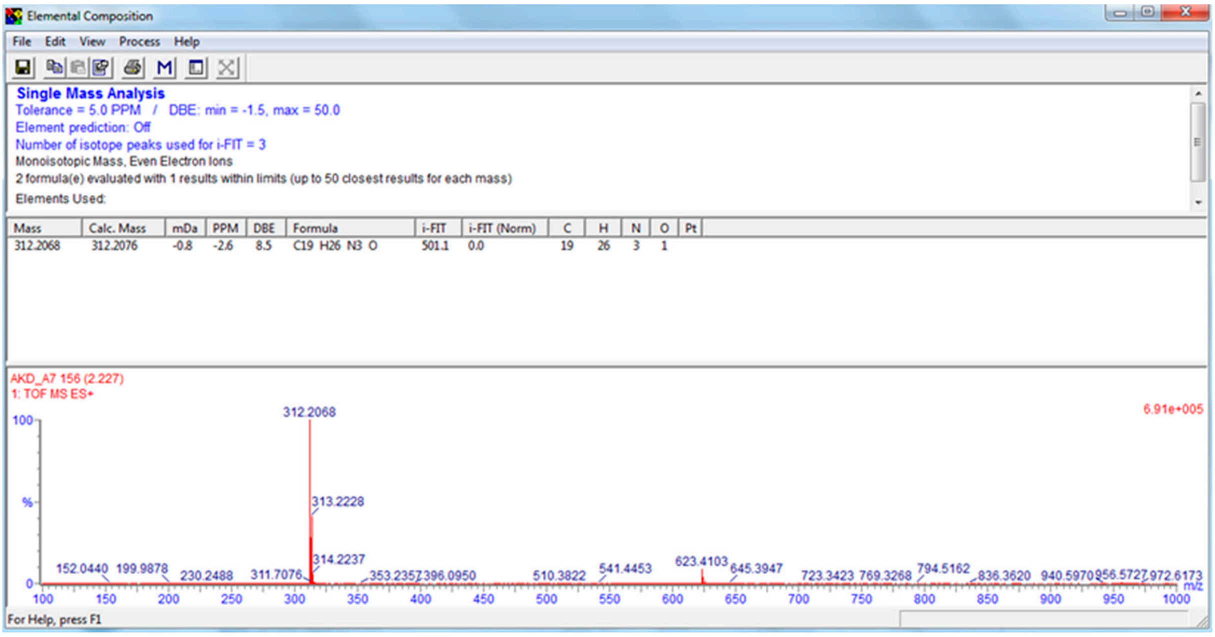

HRMS spectrum of compound 5h

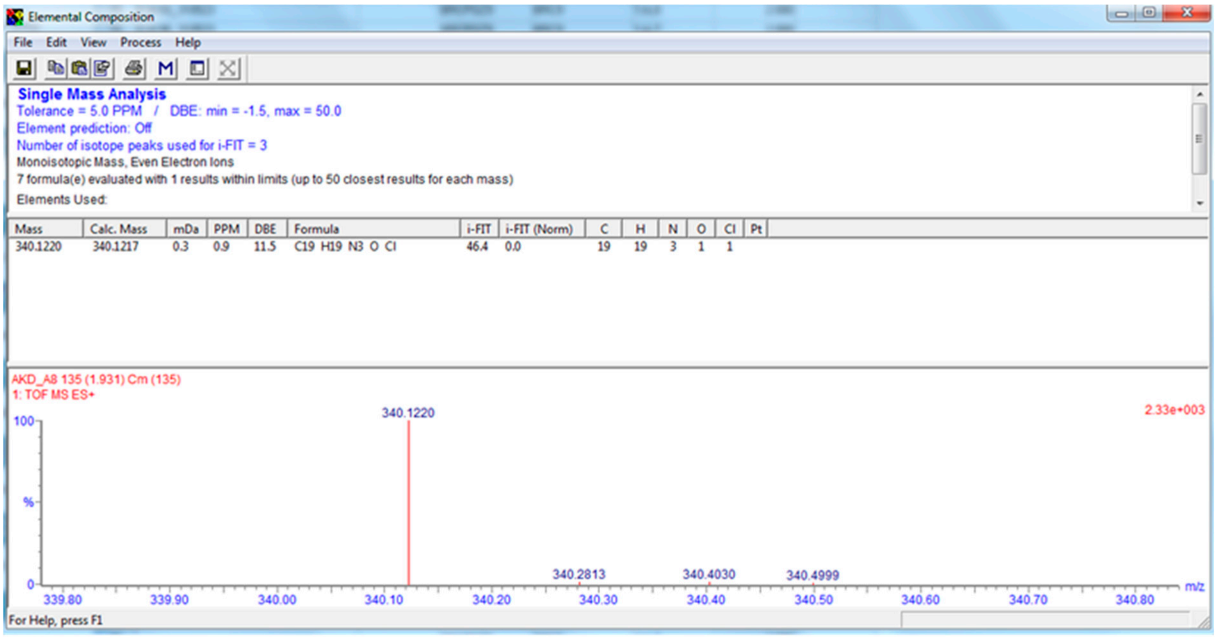

HRMS spectrum of compound 5i

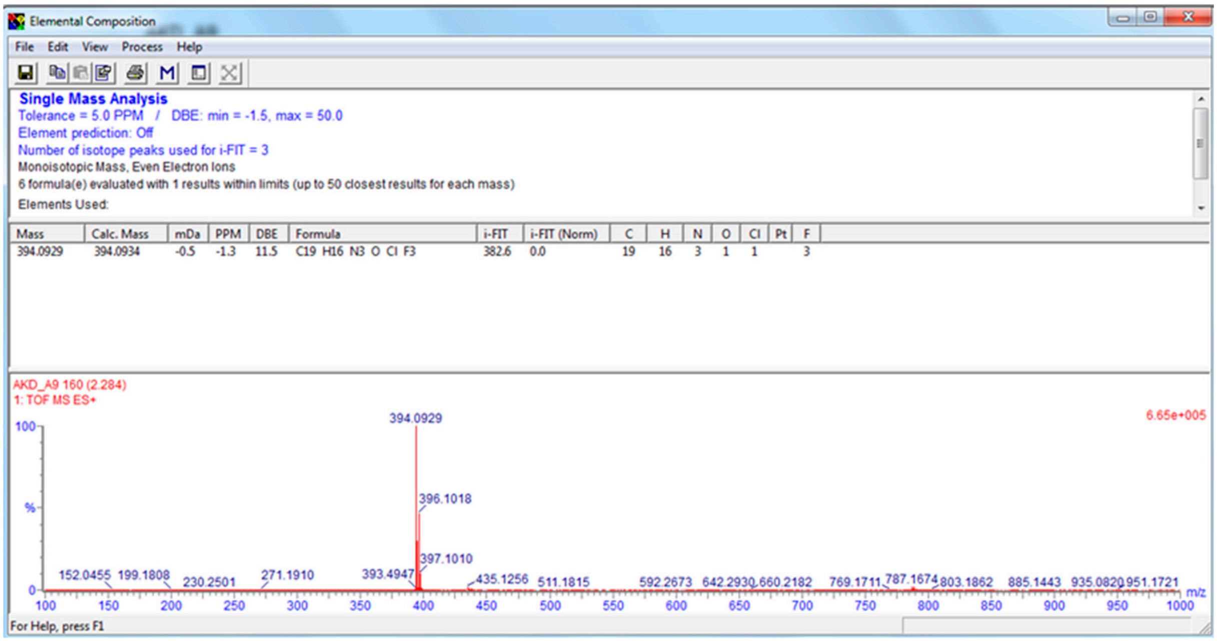

HRMS spectrum of compound 5j

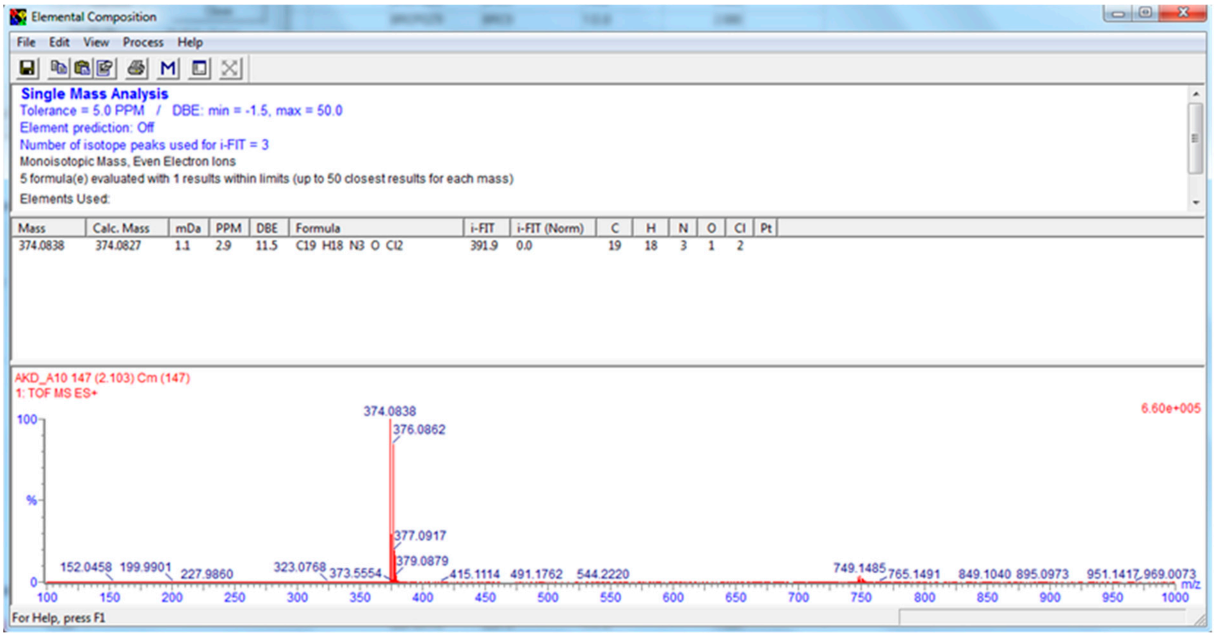

HRMS spectrum of compound 5k

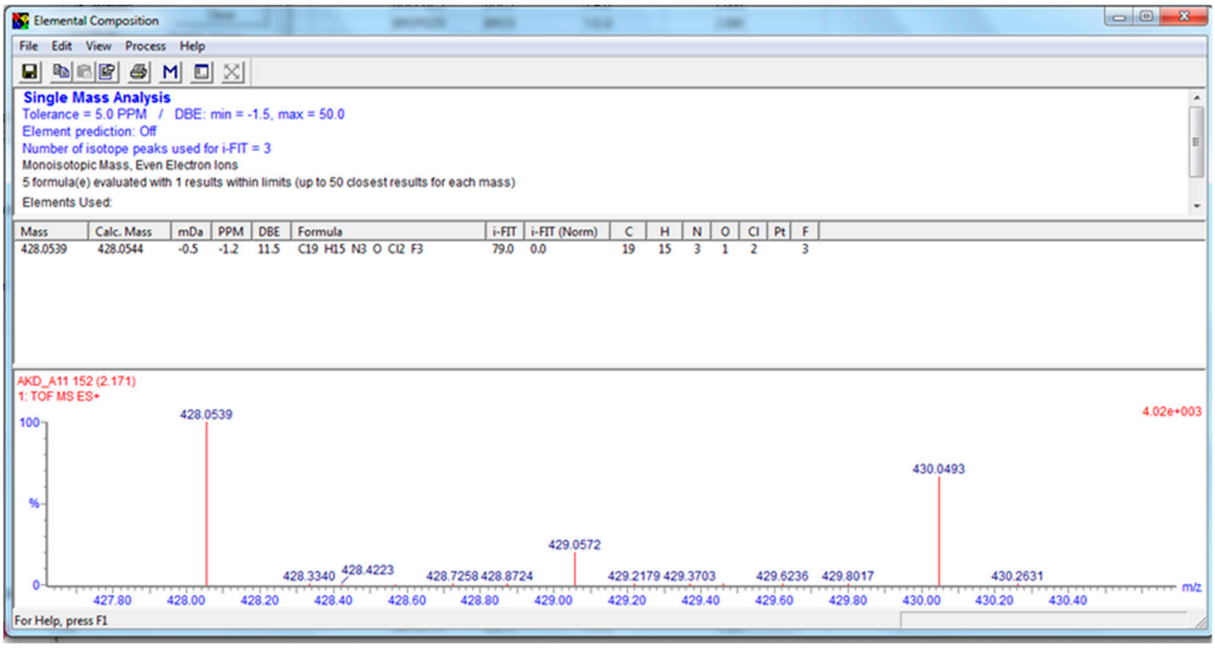
Elemental Composition
File Edit View Process Help
Single Mass Analysis
Tolerance = 5.0 PPM / DBE: min = -1.5, max = 50.0
Element prediction: Off
Number of isotope peaks used for i-FIT = 3
Monoisotopic Mass, Even Electron Ions
5 formula(e) evaluated with 1 results within limits (up to 50 closest results for each mass)
Elements Used:
Mass | Calc. Mass | mDa | PPM | DBE | Formula | i-FIT | i-FIT (Norm) | C | H | N | O | Cl | Pt | F
428.0539 | 428.0544 | -0.5 | -1.2 | 11.5 | C19 H15 N3 O Cl2 F3 | 79.0 | 0.0 | 19 | 15 | 3 | 1 | 2 | | 3
AKD_A11 152 (2.171)
1: TOF MS ES+
4.02e+003
100
%
0
428.0539
430.0493
429.0572
428.3340
428.4223
428.7258 428.8724
429.2179 429.3703
429.6236
429.8017
430.2631
427.80 428.00 428.20 428.40 428.60 428.80 429.00 429.20 429.40 429.60 429.80 430.00 430.20 430.40
m/z
For Help, press F1
